# Gintonin as a Lysophosphatidic Acid-Enriched GPCR Ligand System: Molecular Architecture and Receptor Pharmacology in *Panax ginseng*

**DOI:** 10.3390/biom16030465

**Published:** 2026-03-19

**Authors:** Kyung-Hee Kim, Byong Chul Yoo

**Affiliations:** 1 Department of Applied Chemistry, School of Science and Technology, Kookmin University, Seoul 02707, Republic of Korea; 2 Antibody Research Institute, Kookmin University, Seoul 02707, Republic of Korea; 3 Diagnostic Research Team, InnoBation Bio R&D Center, Seoul 03929, Republic of Korea

**Keywords:** gintonin, lysophosphatidic acid (LPA), LPA receptors, G protein-coupled receptors (GPCRs), lipid–protein complex, calcium signaling, *Panax ginseng*, receptor pharmacology

## Abstract

For decades, the pharmacological identity of *Panax ginseng* has been primarily attributed to triterpenoid saponins known as ginsenosides. However, accumulating evidence indicates that ginseng also contains a structurally distinct lipid–protein complex, termed gintonin, enriched in lysophosphatidic acid (LPA) species. Unlike ginsenosides, which predominantly exert modulatory effects on membrane dynamics and intracellular kinase pathways, gintonin directly activates LPA G protein-coupled receptors (GPCRs), thereby inducing rapid phospholipase C (PLC) activation and intracellular Ca^2+^ mobilization. Biochemical analyses have identified major LPA species within the gintonin fraction, including C16:0, C18:1, and other unsaturated LPA species such as C18:2 stabilized within a proteinaceous matrix that may influence receptor engagement kinetics. Pharmacological studies demonstrate that gintonin preferentially activates LPA_1_ and LPA_3_ receptor subtypes, triggering downstream signaling cascades involving MAPK, PI3K/Akt, and Rho pathways. These receptor-mediated effects occur on a rapid temporal scale, distinguishing gintonin from the slower transcriptional and kinase-modulating actions of ginsenosides. In this review, we synthesize current evidence regarding the chemical architecture, receptor pharmacology, and signaling dynamics of gintonin and propose a dual signaling framework in which steroid-like saponins and lipid GPCR ligands represent complementary molecular axes within *P. ginseng*. Recognition of this layered signaling organization refines the molecular understanding of ginseng biology and highlights gintonin as a unique plant-derived GPCR ligand system.

## 1. Introduction

*Panax ginseng* C.A. Meyer has been used for centuries in traditional medicine and functional nutrition, with its pharmacological activity predominantly attributed to dammarane-type triterpenoid saponins known as ginsenosides [1,2,3]. Ginsenosides exert pleiotropic effects on membrane dynamics, ion channels, intracellular kinase cascades, and transcriptional regulators, including MAPK, PI3K/Akt, and NF-κB signaling pathways [3,4,5,6]. These mechanisms generally involve modulation of intracellular processes and gene expression over extended temporal scales and are consistent with broader observations of lysophospholipid-modulated signaling in mammalian systems [7,8].

In contrast to this steroid-like modulatory paradigm, a distinct bioactive fraction termed gintonin has been identified in *P. ginseng* [9,10]. Gintonin is a glycolipoprotein complex enriched in lysophosphatidic acid (LPA) species that directly engages LPA receptors, a family of G protein-coupled receptors [9,11,12]. Unlike ginsenosides, which do not function as high-affinity receptor agonists, gintonin induces rapid intracellular signaling responses characteristic of canonical GPCR activation [9,13], including G protein-dependent phospholipase C activation and Ca^2+^ mobilization typical of lysophospholipid receptor signaling [8,14].

LPA is a bioactive phospholipid mediator that signals through at least six GPCR subtypes (LPA_1_–LPA_6_), regulating calcium mobilization, cytoskeletal remodeling, proliferation, and migration [12,15,16,17]. Molecular cloning studies established the EDG family as early LPA receptors [14,18], followed by identification of additional non-EDG subtypes including LPA_4_, LPA_5_, and LPA_6_ [15,19,20]. LPA receptors couple to multiple heterotrimeric G proteins, including Gα_q/11, Gα_i/o, and Gα_12/13, thereby activating phospholipase C, IP_3_-mediated Ca^2+^ release, RhoA signaling, PI3K/Akt activation, and MAPK phosphorylation [8,15,17].

The physiological and pathological significance of LPA signaling has been established across neuronal, vascular, immune, and metabolic systems [21,22,23,24]. In the central nervous system, LPA_1_ receptor signaling is required for normal neurodevelopment and myelination [21,25], while aberrant activation contributes to neuropathic pain [22]. In vascular biology, LPA regulates endothelial permeability and RhoA-dependent barrier dysfunction [26,27], and participates in platelet activation and thrombosis [28,29]. LPA receptor signaling also contributes to pulmonary fibrosis [30,31], tumor progression [32,33,34], and metabolic regulation including brown adipose differentiation [35].

Given that GPCRs constitute one of the most pharmacologically exploited receptor families in humans [36,37], and that high-resolution structures have elucidated fundamental mechanisms of receptor activation and biased signaling [38,39,40], modulation of LPA receptor signaling carries substantial translational relevance. Structural resolution of LPA_1_ itself has provided a receptor-level framework for antagonist development and ligand recognition [41].

Biochemical analyses have demonstrated that the gintonin fraction contains specific LPA molecular species, including C16:0 and C18:1, and unsaturated species such as C18:2, stabilized within a proteinaceous scaffold derived from ginseng tissue [9,42]. The Ca^2+^-mobilizing activity of gintonin depends on its LPA content and receptor engagement [9,11], distinguishing it mechanistically from non-receptor-mediated membrane modulators. These findings position gintonin not merely as a secondary metabolite, but as a receptor-targeting lipid complex embedded within a plant matrix.

In mammalian systems, extracellular LPA is tightly regulated by autotaxin-mediated hydrolysis of lysophosphatidylcholine [43,44,45], with structural studies revealing enzymatic mechanisms governing LPA production [46,47]. Lipid phosphate phosphatases further constrain LPA signaling through rapid degradation [48,49,50]. Dysregulation of the autotaxin–LPA axis has been implicated in fibrosis, cancer progression, vascular dysfunction, and metabolic disease [51,52,53,54].

Association of LPA with a protein scaffold, as observed in gintonin, may therefore influence ligand accessibility, receptor engagement thresholds, or signal persistence relative to freely diffusible LPA [12,55]. This raises mechanistic questions regarding ligand presentation and receptor pharmacodynamics in a cross-kingdom signaling context.

Collectively, these mechanistic distinctions are consistent with a dual-axis model of *P. ginseng* biology. The ginsenoside axis operates primarily through modulation of intracellular signaling and membrane-associated processes [3,4], whereas the gintonin axis functions through direct GPCR-mediated lipid signaling [9,13]. The coexistence of delayed kinase modulation and rapid receptor-driven calcium signaling within a single botanical source suggests layered temporal organization rather than biochemical redundancy.

From a molecular pharmacology perspective, this dual architecture reframes *P. ginseng* as an integrated signaling system composed of chemically and mechanistically distinct bioactive entities. Recognition of gintonin as a plant-derived GPCR ligand system invites integration of lipidomics, structural receptor biology, and systems pharmacology approaches to refine the molecular understanding of ginseng bioactivity [24,39,41]. By integrating phytochemistry, receptor pharmacology, and systems-level signaling concepts, this review proposes a dual-axis signaling architecture of *Panax ginseng* and highlights key experimental questions required to establish gintonin as a structurally defined GPCR ligand system.

## 2. Chemical Identity of Gintonin

### 2.1. Discovery and Fractionation

Gintonin was initially isolated from *Panax ginseng* as a non-saponin fraction capable of inducing rapid intracellular Ca^2+^ transients in neuronal cells [9,10]. However, the precise supramolecular organization of gintonin remains incompletely defined. Current models are primarily derived from biochemical fractionation, lipidomic profiling, and proteomic analyses of gintonin-enriched fractions rather than from high-resolution structural characterization [9,42]. Thus, gintonin is presently described operationally as an LPA-enriched lipid–protein complex rather than a structurally resolved molecular entity.

This observation was notable because classical ginseng bioactivity had been primarily attributed to dammarane-type triterpenoid saponins [1,2,3]. Unlike ginsenosides, which are efficiently extracted using aqueous ethanol or methanol and whose amphiphilic steroid-like backbone underlies membrane-associated modulation [4], the Ca^2+^-mobilizing fraction was obtained through sequential aqueous and organic solvent partitioning followed by chromatographic separation [9,42]. This extraction behavior suggested physicochemical properties distinct from classical saponins and more consistent with lipid-associated assemblies.

The rapid Ca^2+^ signaling observed in neuronal cells resembled canonical G protein-coupled receptor (GPCR)-mediated second messenger activation rather than slow transcriptional modulation [8,14]. Subsequent biochemical characterization demonstrated that gintonin is not a single small molecule but a glycolipoprotein complex enriched in lysophosphatidic acid (LPA) species [9,42]. Functional assays showed that this fraction activates LPA receptors in a concentration-dependent manner [9], with signaling kinetics consistent with established LPA receptor activation profiles [12,15,17]. This receptor-dependent mechanism clearly distinguishes gintonin from ginsenosides, which do not function as high-affinity GPCR agonists and instead modulate intracellular pathways indirectly [4,6].

### 2.2. Lysophosphatidic Acid Composition

LPA is a monoacyl glycerophospholipid composed of a glycerol backbone, a single fatty acyl chain, and a phosphate group. In mammalian systems, LPA functions as a potent extracellular signaling lipid that activates at least six GPCR subtypes (LPA_1_–LPA_6_), thereby regulating calcium mobilization, cytoskeletal remodeling, proliferation, and migration [12,15,16,17]. Early molecular cloning studies established the EDG family as LPA receptors [14,18], with additional non-EDG receptors subsequently identified [19,20,56].

Mass spectrometric analyses of the gintonin-enriched fraction revealed the presence of multiple LPA molecular species, comprising multiple LPA species, including C16:0 and C18:1, and unsaturated species such as C18:2, which are considered predominant in recent analyses [9]. These species are also abundant in mammalian lipid signaling contexts [7,55], suggesting biochemical compatibility between plant-derived LPAs and mammalian receptor systems. Importantly, depletion or degradation of LPA components markedly reduces the Ca^2+^-mobilizing activity of the fraction [9,10], consistent with established receptor–ligand dependence observed in LPA signaling [8,14].

In mammalian tissues, extracellular LPA is primarily generated via autotaxin-mediated hydrolysis of lysophosphatidylcholine [43,44,45] and is subject to rapid enzymatic turnover by lipid phosphate phosphatases [48,49,50]. Structural analyses of autotaxin have clarified the enzymatic basis of LPA production [46,47].

In contrast, gintonin-associated LPA appears structurally stabilized within a protein matrix [9,42]. This supramolecular context may influence ligand presentation, receptor accessibility, and signaling kinetics relative to free LPA [12,55], potentially modifying effective local concentration at the receptor interface.

### 2.3. Protein Components and Complex Architecture

Proteomic analyses of the gintonin-enriched fraction have identified several ginseng-derived proteins, including major latex-like proteins and additional proteins with predicted lipid-binding capacity [9,42]. These findings are conceptually consistent with broader observations that plant lipid-binding proteins, such as nonspecific lipid transfer proteins (nsLTPs) and oleosin-associated domains, are capable of forming lipid–protein assemblies through hydrophobic interactions [57,58,59]. In addition, plant phospholipase-related systems contribute to lipid remodeling and signaling contexts that may facilitate lipid–protein association during extraction [60,61,62].

These components appear to associate with lysophosphatidic acid (LPA) species during fractionation, forming a lipid–protein assembly rather than a single defined small molecule entity [9]. However, quantitative stoichiometry and binding specificity have not been comprehensively characterized.

Current biochemical models suggest that LPA molecules are retained within a proteinaceous environment through non-covalent interactions with hydrophobic domains, analogous to lipid-binding modes observed in other lipid-transfer proteins [57,58]. Such association may influence the physicochemical behavior of LPA, including solubility, membrane partitioning, and local concentration at the receptor interface. Nevertheless, direct structural evidence demonstrating defined binding pockets or ordered supramolecular organization is not yet available.

In mammalian systems, extracellular LPA is rapidly metabolized and tightly regulated due to its high biological potency [43,44,49]. Lipid phosphate phosphatases further constrain signaling amplitude and duration [48,50]. A protein-associated configuration in the gintonin fraction may therefore modify effective ligand presentation or stability relative to freely diffusible LPA [12,55].

At present, it remains unclear whether gintonin represents a reproducible supramolecular complex with defined architecture or a heterogeneous lipid–protein aggregate generated during extraction. High-resolution structural characterization of intact gintonin—using approaches such as cryo-electron microscopy, cross-linking mass spectrometry, or integrative modeling—will be necessary to determine whether the observed assembly reflects a biologically organized entity. Structural studies of lipid GPCRs and their complexes provide a conceptual framework for such efforts [38,39,41].

### 2.4. Comparison with Ginsenosides

Ginsenosides are dammarane-type triterpenoid saponins characterized by a rigid steroid-like backbone with variable sugar moieties [1,3]. Their amphiphilic structure allows membrane interaction and modulation of intracellular signaling pathways, including MAPK, PI3K/Akt, and NF-κB cascades [2,4,5,6]. These effects typically occur through membrane modulation and intracellular signaling adaptation rather than direct receptor agonism.

In contrast, gintonin is a lipid-centered signaling complex whose primary bioactive components engage LPA receptors directly [9,10,11]. Canonical LPA receptor activation involves rapid G protein coupling and second-messenger generation [14,15,17], processes that are not characteristic of classical ginsenoside pharmacology.

Whereas ginsenoside-mediated effects often involve transcriptional modulation and delayed kinase signaling [4], gintonin elicits rapid Ca^2+^ transients and GPCR-dependent signaling events [9,13]. This chemical and mechanistic divergence underscores the molecular heterogeneity of *P. ginseng* and supports the dual-axis signaling model.

### 2.5. Processing and Stability Considerations

Processing methods such as steaming, which converts fresh ginseng into red ginseng, significantly alter ginsenoside composition and generate less polar derivatives through deglycosylation and dehydration reactions [1,3]. Such transformations are well documented for saponin constituents.

By contrast, the stability and quantitative variability of gintonin during processing remain less comprehensively characterized [10,42]. Because LPA species are susceptible to hydrolysis and oxidative modification, extraction conditions, storage, and thermal treatment may influence the abundance and integrity of the lipid–protein complex [44,50]. In mammalian systems, circulating LPA levels are tightly regulated by autotaxin activity and degradation enzymes [43,49], highlighting the inherent biochemical lability of this lipid mediator.

Unlike ginsenosides, which are routinely quantified by validated chromatographic methods [3], standardized assays for intact gintonin complexes remain under development. The compositional heterogeneity of the lipid–protein assembly complicates precise quantification and may contribute to variability across preparations [9,42].

### 2.6. Distribution and Specificity

Although LPA is ubiquitous across biological systems [7,12], a reproducible LPA-enriched glycolipoprotein complex with defined GPCR-activating properties has been characterized primarily in *Panax ginseng* [9,10]. Comparable plant-derived LPA–protein assemblies have not been extensively documented in other botanical species.

However, lipid signaling systems in plants are increasingly recognized as important regulatory components [60,63]. Plant phospholipases and lipid-transfer proteins participate in stress responses and developmental processes [61,62,64], suggesting that structured lipid–protein assemblies are not biologically implausible within plant systems.

The apparent specificity of gintonin may therefore reflect limited lipidomic investigation rather than true exclusivity. Compared with saponins and flavonoids, plant-derived lipid mediators remain underexplored. Systematic lipidomic analyses across the genus *Panax* and related species will be required to determine whether gintonin represents a conserved phytolipid signaling system or a species-specific biochemical adaptation.

## 3. Gintonin as a GPCR Ligand System

### 3.1. LPA Receptors: Molecular Background

Lysophosphatidic acid (LPA) receptors belong to the G protein-coupled receptor (GPCR) superfamily and comprise at least six identified subtypes (LPA_1_–LPA_6_), encoded by the genes *LPAR1–6* [14,16,18,19,20,56]. The first cloned LPA receptors were members of the endothelial differentiation gene (EDG) family [14,18], followed by identification of structurally distinct non-EDG receptors, expanding the pharmacological diversity of this receptor group [19,20,56]. Comprehensive nomenclature and functional classification have been summarized by Kihara et al. [16] and Yung et al. [12].

These receptors couple to multiple heterotrimeric G proteins, including Gα_i/o, Gα_q/11, and Gα_12/13, thereby activating diverse downstream signaling cascades such as phospholipase C (PLC), RhoA, MAPK, and PI3K/Akt pathways [8,15,17]. LPA receptor signaling can also engage β-arrestin-mediated pathways and receptor trafficking mechanisms characteristic of GPCR systems [39,40]. Through these mechanisms, LPA signaling regulates calcium mobilization, cytoskeletal remodeling, cell migration, proliferation, and survival [7,12,15].

Physiologically, LPA receptor activity contributes to neuronal development and synaptic plasticity, as demonstrated in LPA_1_-deficient models [21,25]. LPA signaling is required for neuropathic pain initiation [22] and regulates vascular smooth muscle contraction and endothelial barrier dynamics via RhoA-dependent pathways [26,27]. In addition, LPA receptors participate in platelet activation and hemostasis [28,29], immune cell activation [65,66], and fibrotic remodeling in pulmonary injury models [30,31]. LPA signaling has also been implicated in tumor progression, metastasis, and microenvironmental remodeling [32,33,34,52].

In mammalian systems, extracellular LPA is primarily generated through autotaxin-mediated hydrolysis of lysophosphatidylcholine [43,44,45]. Structural analyses of autotaxin have elucidated its catalytic architecture and substrate recognition mechanisms [46,47]. The autotaxin–LPA axis is tightly regulated through rapid enzymatic degradation by lipid phosphate phosphatases [48,49,50], ensuring precise temporal and spatial control of receptor activation. Dysregulation of this axis contributes to fibrosis, cancer, and metabolic disorders [35,51].

The identification of a plant-derived fraction capable of activating mammalian LPA receptors therefore introduced a mechanistically unexpected example of cross-kingdom lipid signaling [9,10]. Within the broader context of GPCR structural biology and receptor pharmacology [37,38,41], gintonin represents an unusual phytochemical entity that directly interfaces with a well-characterized mammalian receptor system. This finding positions gintonin within a defined receptor pharmacology framework rather than within traditional phytochemical paradigms centered on indirect intracellular modulation. An overview of LPA receptor subtypes and downstream signaling pathways is summarized in Table 1.

### 3.2. Direct Activation of LPA Receptors by Gintonin

Functional studies demonstrated that gintonin induces rapid intracellular Ca^2+^ transients in neuronal and non-neuronal cells in a concentration-dependent manner [9,10,11]. These responses resemble canonical LPA receptor-mediated signaling kinetics [8,14]. Pharmacological inhibition experiments showed that Ca^2+^ responses are attenuated by LPA receptor antagonists and by suppression of LPA receptor expression, confirming receptor-mediated activation [9,15].

In contrast, ginsenosides do not elicit comparable rapid Ca^2+^ mobilization via LPA receptors and instead modulate intracellular pathways indirectly through membrane interactions or kinase regulation [2,4,6], underscoring a clear mechanistic divergence between these two ginseng-derived bioactive classes.

Pharmacological profiling suggests that gintonin preferentially engages LPA_1_ and LPA_3_ receptor subtypes in several cellular systems [9,17]. Although receptor activation by gintonin has been demonstrated in several cellular systems [9,10,11], quantitative pharmacological characterization remains limited. Reported Ca^2+^ mobilization assays indicate that gintonin fractions can induce responses in the low micromolar range when normalized to LPA-equivalent concentrations, broadly comparable to reported EC50 values for free LPA in similar systems [8,15]. However, direct comparisons between intact gintonin preparations and defined LPA species under controlled molar conditions remain scarce. Further pharmacological analyses—including subtype-specific receptor assays and concentration–response comparisons—will be required to determine whether gintonin-associated LPA differs in potency or efficacy relative to freely diffusible LPA. These receptor subtypes are highly expressed in the central nervous system and vascular tissues [15,21,25], aligning with reported neuroactive and vasomodulatory properties attributed to gintonin [11]. LPA_1_ and LPA_3_ receptors are also implicated in fibrosis and tissue remodeling [30,67], suggesting that subtype-selective engagement may carry functional significance.

### 3.3. Intracellular Signaling Dynamics

Upon receptor engagement, gintonin stimulates Gα_q/11-mediated activation of phospholipase C (PLC), leading to inositol trisphosphate (IP_3_) production and subsequent Ca^2+^ release from intracellular stores [8,9,14]. This rapid second-messenger response occurs within seconds to minutes and represents a hallmark of GPCR activation [38,39]. Such kinetics sharply contrast with the slower genomic and kinase-modulating mechanisms often associated with ginsenosides [4,5].

Downstream consequences of gintonin-induced Ca^2+^ mobilization include activation of Ca^2+^/calmodulin-dependent kinases and phosphorylation of ERK/MAPK pathways, consistent with established LPA receptor signaling cascades [15,17]. LPA receptor activation can also engage PI3K/Akt and RhoA-dependent cytoskeletal remodeling pathways [8,26].

**Figure 1:**
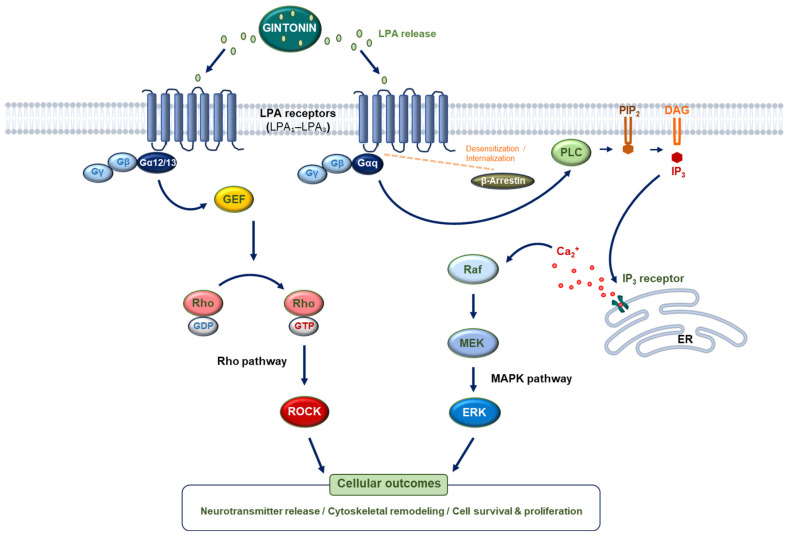
Proposed receptor-mediated signaling cascade triggered by gintonin. Gintonin-associated LPA species bind to LPA receptors (primarily LPA_1_–LPA_3_), leading to activation of heterotrimeric G proteins. Gαq/11-mediated stimulation of phospholipase C (PLC) generates inositol trisphosphate (IP_3_), resulting in intracellular Ca^2+^ release. Additional signaling branches may include activation of MAPK, PI3K/Akt, and RhoA pathways. The rapid kinetics of this cascade contrast with the slower modulatory effects typically attributed to ginsenosides.

In neuronal systems, LPA receptor activation regulates synaptic function and excitability [21,25], and gintonin has been reported to enhance neurotransmitter release in a Ca^2+^-dependent manner [11]. These observations support the interpretation of gintonin as a receptor-targeting ligand complex rather than a nonspecific membrane modulator. The proposed receptor-mediated signaling cascade is illustrated in Figure 1.

### 3.4. Receptor Bias and Signaling Specificity

An important unresolved question is whether gintonin elicits signaling responses quantitatively and kinetically equivalent to those induced by freely diffusible endogenous LPA, or whether presentation of LPA within a protein-associated scaffold modifies receptor-level signaling output.

In contemporary GPCR pharmacology, ligand bias (functional selectivity) refers to the ability of structurally distinct ligands acting at the same receptor to preferentially activate specific downstream pathways—such as G protein-mediated signaling versus β-arrestin recruitment [39]. Structural studies have demonstrated that distinct ligand–receptor interaction geometries can stabilize alternative receptor conformations, thereby altering downstream coupling profiles [38,68].

At first glance, gintonin-associated LPA and endogenous LPA share identical chemical headgroups and therefore might be expected to behave equivalently at orthosteric receptor binding sites. However, ligand bias does not necessarily require chemical novelty at the headgroup level. It may also arise from differences in ligand presentation, membrane partitioning, local concentration dynamics, or residence time at the receptor interface.

Several theoretical mechanisms by which gintonin could modify signaling topology can be considered.

#### 3.4.1. Presentation-Dependent Bias

Because gintonin delivers LPA within a protein-associated complex, ligand access to the orthosteric binding pocket may be spatially constrained or locally concentrated. This configuration could alter the effective on-rate (k_on) and dissociation kinetics (k_off), as well as modify the local membrane microenvironment during receptor engagement. Such differences may potentially produce kinetic bias, in which temporal aspects of receptor activation—rather than static binding affinity—determine downstream pathway selection.

#### 3.4.2. Membrane Partition-Dependent Signaling

LPA receptors reside within lipid bilayers, and GPCR function is sensitive to membrane microdomain composition [69]. If the gintonin scaffold modifies LPA insertion into the membrane or alters lipid raft localization, receptor clustering or dimerization dynamics may be affected.

Because GPCR signaling efficiency can depend on receptor density and microdomain confinement [37], altered membrane partitioning may indirectly bias signaling output even if orthosteric binding is chemically identical.

#### 3.4.3. Ligand Residence Time and Signal Persistence

Emerging pharmacological models emphasize ligand residence time as a determinant of signaling bias [70]. If protein association transiently shields LPA from rapid enzymatic degradation or increases receptor rebinding probability, signaling duration may be prolonged relative to endogenous LPA. Sustained receptor engagement can favor β-arrestin recruitment, receptor internalization, or endosomal signaling pathways distinct from rapid plasma membrane-restricted signaling [71]. Thus, even identical ligand chemistry may produce different pathway outputs depending on kinetic context.

#### 3.4.4. Endosomal Versus Plasma Membrane Signaling Topology

Recent GPCR research demonstrates that receptor signaling is not confined to the plasma membrane; endosomal GPCRs can generate spatially distinct signaling cascades [39,71]. If gintonin alters receptor internalization dynamics—either by modifying activation amplitude or by influencing desensitization kinetics—downstream transcriptional consequences could differ from those triggered by freely diffusible LPA.

#### 3.4.5. Current Evidence and Limitations

At present, available data primarily demonstrate Ca^2+^ mobilization and G protein-dependent signaling following gintonin exposure [9]. However, quantitative bias factors have not been calculated, β-arrestin recruitment assays have not been systematically performed, receptor internalization kinetics have not been directly compared with endogenous LPA, and real-time kinetic signaling profiles have not been resolved using pathway-selective biosensors. Therefore, while receptor activation is well supported, formal classification of gintonin as a biased ligand remains premature.

#### 3.4.6. Conceptual Implication

The key mechanistic question is not whether gintonin activates LPA receptors—it clearly does—but whether its mode of ligand delivery modifies receptor conformational landscapes or signaling kinetics in a manner that distinguishes it pharmacodynamically from endogenous LPA.

Should future quantitative analyses demonstrate quantitative differences in pathway coupling or signaling persistence, gintonin would represent not merely a plant-derived source of LPA, but a presentation-modified GPCR ligand system with distinct pharmacological properties.

Until such analyses are performed, gintonin should be regarded as a receptor-activating LPA delivery complex whose potential for signaling bias remains an open and experimentally tractable question. A conceptual comparison between endogenous LPA and gintonin-associated LPA is summarized in Table 2.

### 3.5. Endogenous LPA Versus Gintonin-Derived LPA

Endogenous LPA operates within tightly regulated mammalian lipid signaling networks characterized by rapid synthesis and degradation [43,44,46]. In contrast, gintonin introduces LPA in a preassembled lipid–protein complex form [9,42]. This distinction may influence receptor activation thresholds, signal duration, and desensitization kinetics.

Endogenous LPA is rapidly degraded by lipid phosphate phosphatases [48,49,50], limiting signal persistence. Association with a protein scaffold may transiently shield LPA from enzymatic turnover and modify ligand presentation at the receptor interface [55].

Sustained LPA receptor signaling has been implicated in pathological contexts such as fibrosis [30,31], vascular remodeling [26], thrombosis [29], and tumor progression [32,33,34]. Understanding whether gintonin-induced signaling differs quantitatively from endogenous LPA exposure is therefore critical for evaluating pharmacological relevance.

### 3.6. Functional Implications for Ginseng Pharmacology

The capacity of gintonin to directly activate GPCR-mediated second-messenger systems introduces a rapid signaling axis within *Panax ginseng* pharmacology [9,10]. Whereas ginsenosides predominantly influence intracellular kinase cascades, membrane properties, and transcriptional programs over extended timescales [2,3,4], gintonin engages receptor-driven Ca^2+^ signaling within seconds.

This temporal and mechanistic complementarity supports a dual-axis model of ginseng bioactivity: a steroid-like modulatory axis mediated by triterpenoid saponins and a receptor-targeting lipid axis mediated by gintonin [9,13]. Such layered signaling architecture may help reconcile the broad spectrum of physiological effects historically attributed to ginseng but incompletely explained by saponins alone.

## 4. Dual Signaling Architecture of *Panax ginseng*

### 4.1. Reconsidering the Ginsenoside-Centric Paradigm

For decades, the biological identity of *Panax ginseng* has been interpreted primarily through the pharmacology of ginsenosides. These dammarane-type triterpenoid saponins exhibit anti-inflammatory, neuroprotective, metabolic, and anti-proliferative effects across diverse experimental systems [1,2,3]. Their regulatory effects extend to cancer biology and inflammatory signaling pathways, including NF-κB and PI3K/Akt modulation [5,6].

Mechanistically, ginsenosides interact with lipid membranes and membrane microdomains, influencing ion channel activity and intracellular signaling cascades [4]. These compounds regulate kinase pathways such as MAPK and PI3K/Akt and may influence nuclear receptor-mediated transcriptional programs [2,3]. However, ginsenosides generally do not function as high-affinity, receptor-selective agonists for defined GPCR subtypes.

Instead, their actions are typically modulatory, altering cellular responsiveness and membrane signaling tone rather than initiating rapid second-messenger cascades. This pharmacological profile aligns with the historical classification of ginseng as an “adaptogen,” a concept referring to broad regulatory activity that enhances systemic resilience without targeting a single receptor system [3].

The identification of gintonin as a direct agonist of LPA receptors introduces a mechanistically distinct signaling modality within *P. ginseng* [9,10]. Rather than operating through indirect modulation alone, gintonin engages defined GPCR subtypes and induces rapid intracellular Ca^2+^ mobilization [9,11], consistent with canonical LPA receptor activation dynamics [15,17]. This distinction challenges reductionist interpretations that attribute ginseng pharmacology exclusively to triterpenoid saponins.

### 4.2. Two Molecular Axes: Steroid-like Modulation Versus Lipid-GPCR Signaling

The coexistence of ginsenosides and gintonin within *P. ginseng* suggests the presence of two chemically and mechanistically distinct molecular axes (Table 3). The first axis is defined by ginsenosides, steroid-like triterpenoid saponins characterized by a rigid dammarane backbone and variable glycosylation patterns [1,3]. Their primary actions involve modulation of membrane properties and indirect regulation of intracellular kinase cascades [4,5]. These effects typically manifest over longer temporal scales and often require transcriptional reprogramming.

The second axis is defined by gintonin, a lipid-centered glycolipoprotein complex enriched in LPA species [9,42]. Its primary mode of action is direct activation of LPA GPCR subtypes—particularly LPA_1_–LPA_3_ [14,17]—resulting in PLC/IP_3_-mediated Ca^2+^ mobilization and rapid second-messenger signaling [8,38].

From a receptor pharmacology perspective, this distinction reflects two fundamentally different signaling topologies. The ginsenoside axis operates primarily through membrane-associated modulation and intracellular pathway tuning, whereas the gintonin axis initiates ligand–receptor coupling at the plasma membrane, engaging heterotrimeric G proteins and second-messenger systems [15,39]. Structural studies of GPCR activation underscore how defined ligand–receptor interactions can trigger rapid conformational changes and downstream signaling specificity [38,41].

These axes differ not only in chemical structure but also in temporal dynamics and systems-level integration. Whereas the ginsenoside axis predominantly influences intracellular regulatory networks, the gintonin axis provides a receptor-driven excitatory signaling component. The coexistence of these modalities within a single botanical source suggests layered pharmacological architecture rather than biochemical redundancy.

### 4.3. Systems-Level Integration and Translational Implications

The translational relevance of the dual-axis model becomes clearer when situated within the broader biological and clinical landscape of lysophosphatidic acid (LPA) signaling. Unlike many phytochemical targets, LPA receptors are embedded in a well-characterized signaling network that regulates fibrosis, tumor progression, vascular remodeling, immune cell trafficking, and metabolic homeostasis [16,17,55]. This established framework provides a mechanistic context for interpreting gintonin-mediated receptor activation. An important translational consideration concerns whether concentrations of gintonin achievable in vivo are sufficient to activate LPA receptors. While several cellular studies demonstrate robust Ca^2+^ responses following exposure to purified gintonin fractions [9,10,11], quantitative measurements of systemic gintonin levels following oral or parenteral administration remain limited. Future pharmacokinetic studies measuring circulating LPA species and receptor occupancy will therefore be important for evaluating the physiological relevance of gintonin-mediated signaling.

#### 4.3.1. Fibrosis and Tissue Remodeling

LPA receptor activation, particularly via LPA_1_, plays a central role in tissue injury responses and fibrotic remodeling. Genetic deletion or pharmacological inhibition of LPA_1_ attenuates bleomycin-induced pulmonary fibrosis, demonstrating a causal link between receptor signaling and fibroblast recruitment [30]. Subsequent studies have shown that LPA promotes fibroblast chemotaxis, epithelial barrier dysfunction, and myofibroblast differentiation, reinforcing its role as a pro-fibrotic mediator [31,72].

Because fibrotic remodeling is highly sensitive to signaling duration and receptor engagement kinetics, even quantitative differences in ligand presentation may alter downstream outcomes. In this context, the protein-associated configuration of gintonin-derived LPA raises mechanistic questions regarding signal persistence and receptor desensitization relative to freely diffusible endogenous LPA [43,50]. Thus, fibrosis represents not merely a disease example but a systems-level readout of sustained LPA signaling intensity.

#### 4.3.2. Cancer: Migration, Microenvironment, and Metastatic Signaling

LPA signaling is strongly implicated in cancer cell migration, invasion, and metastatic progression. In ovarian carcinoma, peritoneal mesothelial cells generate LPA that enhances tumor cell adhesion and invasion [32]. Overexpression of specific LPA receptor subtypes increases tumorigenicity and metastatic potential [33]. Hypoxic microenvironments further amplify LPA-driven signaling, linking lipid mediators to stress adaptation in tumors [73].

LPA_2_ deletion reduces tumor formation in inflammatory carcinogenesis models, underscoring receptor subtype-specific contributions [74]. Additional pathway-level evidence demonstrates that LPA-induced invasion involves VEGFR-2 transactivation and downstream ERK activation [75].

Within this oncology framework, gintonin-mediated receptor activation should be interpreted through receptor pharmacology rather than through generalized “anticancer” narratives. Critical translational variables include receptor subtype engagement, signaling bias, ligand concentration dynamics, and tissue-specific receptor expression patterns [17,70].

#### 4.3.3. Metabolic Regulation and Energy Signaling

The autotaxin–LPA axis also intersects with metabolic regulation. Autotaxin-derived LPA suppresses brown adipose differentiation and promotes diet-induced obesity in murine models [35]. LPA receptor signaling influences PI3K/Akt pathways and intracellular calcium dynamics, both central to metabolic flux and insulin responsiveness [15,17].

These findings position LPA signaling as a programmable endocrine-like lipid system. In this context, gintonin-mediated receptor activation may influence metabolic sensitivity or differentiation trajectories, particularly if ligand presentation differs in magnitude or duration from endogenous LPA generation [43].

#### 4.3.4. Vascular Biology and Barrier Function

LPA receptors regulate vascular smooth muscle contraction, endothelial permeability, and cytoskeletal organization. Early studies demonstrated that LPA modulates endothelial barrier properties via RhoA-dependent mechanisms [26]. LPA accumulates in atherosclerotic plaques and activates platelets, implicating the pathway in vascular pathology [28].

Autotaxin and LPA also regulate murine hemostasis and thrombosis [29]. More recently, LPA_6_ has been implicated in blood–brain barrier integrity [76]. These observations underscore the role of receptor-defined lipid signaling in vascular and barrier homeostasis.

Given that gintonin engages LPA receptors directly [9], its vascular effects should be interpreted within this established signaling paradigm rather than as nonspecific phytochemical activity.

#### 4.3.5. Immune Cell Dynamics and Inflammatory Recruitment

LPA receptors are expressed on lymphocytes and monocytes, where they regulate calcium-dependent activation and migration [65,66]. LPA induces NF-κB-dependent cytokine expression in epithelial systems, linking receptor signaling to inflammatory recruitment [77].

These findings suggest that rapid GPCR-mediated Ca^2+^ signaling may represent an early-stage immune regulatory event, whereas slower kinase- and transcription-based mechanisms—often attributed to ginsenosides—may shape later inflammatory tone. The temporal layering inherent in the dual-axis model therefore aligns with immune system signaling hierarchies.

#### 4.3.6. Drug Discovery Perspective

The translational significance of LPA receptor biology is further underscored by drug discovery efforts targeting both receptors and autotaxin. Structure-based GPCR drug design has transformed receptor pharmacology [37,38]. Biased agonism and pathway-selective signaling are now recognized as central determinants of therapeutic efficacy [70].

Autotaxin inhibitors have been developed as non-lipid small molecules to modulate LPA production [78], reflecting the therapeutic maturity of this signaling axis.

Within this framework, gintonin can be conceptualized as a plant-derived ligand system operating within a clinically validated GPCR network. The key translational questions are therefore quantitative and pharmacodynamic: receptor subtype selectivity, signaling bias, ligand stability, tissue distribution, and desensitization kinetics.

#### 4.3.7. Integrative Interpretation

Taken together, fibrosis, cancer progression, metabolic remodeling, vascular regulation, and immune signaling converge on LPA receptor activation as a shared mechanistic node [16,17]. Because gintonin directly engages this receptor family [9], its biological interpretation is strengthened by alignment with a mature signaling literature.

Importantly, this does not imply that gintonin recapitulates pathological LPA signaling. Rather, it situates the lipid–GPCR axis within a quantitatively defined pharmacological space. When integrated with the slower, modulatory effects of ginsenosides [3,4], the result is a temporally layered signaling architecture that may account for the breadth of physiological effects historically attributed to *Panax ginseng*.

### 4.4. Complementarity or Redundancy

Whether these two axes function independently, redundantly, or synergistically remains incompletely defined. Direct experimental interrogation of pathway cross-talk is limited; however, several mechanistic scenarios can be considered.

One possibility is parallel independence, in which ginsenosides and gintonin activate distinct molecular targets without direct interaction, resulting in additive physiological effects [3,9].

Alternatively, sequential modulation may occur: gintonin initiates rapid GPCR-mediated signaling through defined LPA receptor pathways [15,17], while ginsenosides subsequently modulate downstream kinase cascades or transcriptional programs such as PI3K/Akt and NF-κB [5,6], reinforcing or refining the initial response.

A third possibility involves membrane microdomain interactions. Ginsenosides influence membrane fluidity and lipid raft organization [4]. Because GPCR localization, dimerization, and signaling efficiency are sensitive to membrane microenvironment [37,69], it is conceivable that ginsenosides indirectly modulate gintonin-mediated receptor signaling at the membrane level.

Although these models remain speculative, the coexistence of two chemically distinct bioactive systems argues against simple redundancy and instead suggests potential functional complementarity.

### 4.5. Temporal and Systems-Level Integration

From a systems biology perspective, the temporal distinction between rapid GPCR activation and slower transcriptional modulation is particularly noteworthy. GPCR-mediated Ca^2+^ signaling occurs within seconds to minutes following receptor engagement [14,38,79], whereas transcriptional and kinase-modulatory effects often require extended time frames [4,5].

In neuronal contexts, rapid Ca^2+^ mobilization influences excitability, synaptic transmission, and short-term plasticity [21,25], while longer-term transcriptional regulation may contribute to structural remodeling and neuroprotection. In vascular tissues, acute LPA receptor activation regulates endothelial permeability and RhoA-dependent cytoskeletal dynamics [26,27], whereas saponin-mediated signaling may modulate inflammatory and remodeling responses over prolonged periods [3].

This layered temporal organization resembles hierarchical signaling models described in GPCR biology, where rapid membrane-proximal events precede transcriptional reprogramming [39,40]. The coexistence of these mechanisms within a single phytochemical matrix supports a hierarchical rather than redundant signaling architecture.

### 4.6. Implications for Standardization and Analytical Strategy

Quality control of ginseng preparations has historically focused on quantification of selected ginsenosides as markers of potency [1,3]. Regulatory standards in several jurisdictions specify minimum concentrations of particular saponins for classification and commercial labeling.

If gintonin represents a mechanistically distinct signaling axis [9,10], reliance solely on ginsenoside quantification may provide an incomplete assessment of biological activity. Variability in gintonin content across cultivation conditions, processing methods, or extraction protocols [42] could contribute to differences in pharmacological outcomes.

Because LPA species are enzymatically labile and subject to rapid metabolism [43,49], analytical strategies incorporating lipidomic profiling and protein-associated lipid characterization may be necessary. Advances in structural lipid–protein analysis [57,58] and receptor structural biology [41] provide conceptual tools for refining such strategies.

### 4.7. Toward an Integrated Molecular Model

Collectively, available evidence is consistent with a conceptual framework in which *Panax ginseng* contains at least two major signaling systems: a steroid-like triterpenoid axis mediated by ginsenosides [3,4] and a lipid GPCR-targeting axis mediated by gintonin [9,11]. These systems operate through distinct yet potentially complementary pathways.

Recognition of this dual architecture aligns with contemporary receptor pharmacology, which emphasizes ligand-specific signaling dynamics and systems-level integration [37,39]. This model situates ginsenosides within a broader molecular ecosystem in which receptor-driven lipid signaling coexists with triterpenoid-mediated modulation.

## 5. System-Level Biological Effects: A Process-Oriented Interpretation

Rather than interpreting the biological effects of *Panax ginseng* through discrete disease categories, the dual-axis model can be more coherently understood through conserved physiological processes regulated by LPA receptor signaling. Because LPA receptors are broadly expressed across neuronal, vascular, immune, and metabolic tissues [16,17], gintonin-mediated receptor activation is positioned to influence fundamental regulatory mechanisms rather than isolated pathological states.

### 5.1. Rapid Calcium Dynamics and Excitability Control

Calcium signaling serves as a universal intracellular second messenger governing excitability, secretion, cytoskeletal remodeling, and transcriptional priming. LPA receptor activation via Gαq/11 stimulates phospholipase C, generating IP_3_ and triggering rapid Ca^2+^ release from endoplasmic reticulum stores [15,17].

In neuronal systems, LPA receptors regulate neurite retraction, synaptic transmission, and network excitability [16,55]. Rapid Ca^2+^ microdomain dynamics at presynaptic Ca(V)2.1 channels critically shape neurotransmitter release probability and short-term synaptic plasticity [80].

Gintonin-induced Ca^2+^ mobilization [9,11] therefore aligns with canonical GPCR-driven excitatory signaling. These kinetics contrast sharply with the slower genomic and kinase-mediated modulation attributed to ginsenosides [4,6].

From a systems perspective, gintonin may operate primarily at the level of acute signal integration, whereas ginsenosides influence longer-term adaptive transcriptional states.

### 5.2. Cytoskeletal Remodeling and Cell Motility Programs

LPA receptor activation engages Gα12/13–RhoA signaling pathways, regulating actin dynamics, focal adhesion turnover, and cell motility [15,17]. These processes underlie neuronal growth cone behavior, endothelial barrier regulation, and immune cell migration.

Cytoskeletal reorganization is a rapid-response module within multicellular systems. Because GPCR-mediated Rho activation occurs within seconds to minutes, the gintonin axis may influence cell positioning and barrier responsiveness in a temporally constrained manner.

By contrast, ginsenosides have been reported to modulate inflammatory transcription factors such as NF-κB and MAPK cascades [5,6], which operate over longer time scales. The dual-axis model thus reflects a distinction between rapid structural reconfiguration (lipid–GPCR axis) and slower regulatory recalibration (triterpenoid axis).

### 5.3. Energy Signaling and Metabolic Responsiveness

Intracellular calcium and PI3K/Akt pathways intersect with metabolic control networks [15]. LPA receptor signaling modulates glucose metabolism and adipocyte differentiation [35]. Autotaxin–LPA signaling influences systemic metabolic phenotypes in diet-induced models [35,50].

Calcium-dependent kinases also regulate mitochondrial function and metabolic flux [80]. Therefore, rapid receptor-mediated Ca^2+^ signaling may transiently alter metabolic sensitivity. Ginsenosides, conversely, have been linked to modulation of insulin signaling pathways and AMPK-related metabolic regulation [3,5]. In combination, the two axes may represent layered metabolic control: receptor-initiated signaling sensitivity followed by transcriptional and kinase-mediated stabilization.

### 5.4. Vascular Tone and Barrier Regulation

LPA receptors in vascular smooth muscle and endothelial cells regulate contraction, permeability, and cytoskeletal tension [17,26]. LPA accumulation in vascular lesions underscores its physiological relevance [28].

Acute GPCR-mediated Ca^2+^ signaling influences vascular tone at short time scales, whereas longer-term endothelial remodeling involves transcriptional reprogramming and inflammatory signaling cascades.

Ginsenosides have been reported to modulate nitric oxide production and endothelial protective pathways [2,4]. Thus, gintonin may participate in immediate receptor-driven vascular adjustments, while ginsenosides influence sustained endothelial homeostasis.

### 5.5. Immune Activation and Signaling Hierarchies

Immune cells express functional LPA receptors that regulate calcium flux, migration, and cytokine expression [65,66]. LPA can activate NF-κB-dependent transcriptional programs in epithelial cells [77].

Immune signaling hierarchies typically involve rapid receptor-triggered events followed by transcriptional reinforcement. In this framework, gintonin-mediated GPCR activation may represent an early signaling layer, whereas ginsenoside-mediated modulation of NF-κB and MAPK may contribute to later-stage inflammatory regulation [6].

The temporal layering observed across neuronal, metabolic, vascular, and immune contexts reinforces the interpretation of the dual-axis architecture as a conserved organizational principle.

### 5.6. Temporal Layering as a Systems Organizing Principle

Across multiple physiological domains, a consistent temporal stratification of signaling responses becomes apparent. Rapid GPCR-driven events—occurring within seconds to minutes—include Ca^2+^ mobilization, Rho activation, and cytoskeletal remodeling [15,17]. In contrast, kinase modulation, transcriptional regulation, and inflammatory reprogramming unfold over hours to days [3,6]. Contemporary systems biology frameworks emphasize that such networks are hierarchically organized in time, whereby early second-messenger pulses condition subsequent genomic programs [80].

In this context, gintonin and ginsenosides may constitute temporally layered regulatory modules within the same phytochemical system. Table 3 summarizes this dual-axis organization at a systems level, reframing *Panax ginseng* not as a collection of isolated bioactivities but as a temporally integrated signaling architecture.

## 6. Unresolved Questions

### 6.1. Structural Definition and Molecular Uniformity

Gintonin has been described as an LPA-enriched glycolipoprotein complex based on chromatographic fractionation, lipidomic profiling, and proteomic identification [9,42]. Nevertheless, its supramolecular organization has not been resolved with structural precision. Fundamental parameters—including stoichiometry, minimal receptor-active configuration, structural reproducibility, physiological stability, and the existence of defined lipid-binding domains—remain undefined.

Accordingly, it is uncertain whether gintonin constitutes a structurally coherent lipid–protein assembly or represents a heterogeneous extraction-derived association in which bioactivity is driven by variably presented LPA molecules. This distinction is central rather than peripheral. Within receptor pharmacology, ligand identity requires structural reproducibility; absent such definition, observed receptor activation may reflect concentration-dependent LPA delivery rather than the action of a discrete supramolecular ligand system.

Furthermore, the possibility that free LPA—released during preparation or dilution—constitutes the actual receptor-activating species has not been rigorously excluded. Without quantitative assessment of dissociation dynamics, protein-bound versus free LPA fractions, and receptor engagement kinetics, the degree to which the protein scaffold modifies pharmacodynamics remains uncertain.

Lipid–protein complexes frequently exhibit structural plasticity dependent on ionic strength, pH, temperature, and solvent exposure [55,81]. Therefore, extraction-driven aggregation cannot be excluded without orthogonal structural validation.

High-resolution approaches—such as cryo-electron microscopy of intact assemblies, native mass spectrometry to determine stoichiometry, cross-linking mass spectrometry to map lipid–protein interfaces, or hydrogen–deuterium exchange assays to assess conformational stability—have not yet been applied. In the absence of such structural resolution, the molecular identity of gintonin remains operational rather than structurally defined. Thus, structural resolution is not a refinement exercise but a prerequisite for mechanistic classification.

Determining whether gintonin represents a structurally stable supramolecular ligand complex, a protein-facilitated lipid delivery system, or a heterogeneous extraction artifact is critical for establishing its legitimacy as a discrete pharmacological entity rather than as a preparation-dependent LPA-containing fraction.

Until these structural uncertainties are resolved, mechanistic interpretation of gintonin signaling must be considered provisional at the level of molecular architecture. Key structural uncertainties and experimental priorities are summarized in Table 4.

### 6.2. Receptor Bias and Signaling Selectivity

Although gintonin activates LPA receptors and induces Ca^2+^ mobilization, the extent to which it exhibits signaling bias relative to endogenous LPA remains unknown. In GPCR biology, structurally distinct ligands acting at the same receptor can preferentially activate specific G protein pathways or β-arrestin-mediated signaling branches [70].

Whether gintonin preferentially engages Gαq versus Gαi pathways, alters β-arrestin recruitment, or modifies receptor desensitization kinetics has not been systematically examined. GPCR internalization and endosomal signaling can significantly alter downstream pathway output [71]. Comprehensive pharmacological profiling using pathway-specific reporters and receptor subtype-selective systems would clarify whether gintonin functions as a biased ligand or primarily mimics endogenous LPA signaling [36].

### 6.3. Biosynthetic Origin in Plants

The biosynthetic origin of LPA accumulation within *P. ginseng* tissues remains insufficiently characterized. In mammalian systems, extracellular LPA production is closely linked to autotaxin activity and phospholipid metabolism [43,50].

In plants, phospholipase-mediated lipid remodeling and stress-induced phospholipid signaling pathways are well documented in defense and abiotic stress responses [60,61,62,63]. However, the specific enzymatic contributors to LPA accumulation in ginseng have not been defined. Whether formation of the gintonin complex is biologically regulated or primarily extraction-driven remains unclear.

### 6.4. Quantification and Standardization

Current quality control standards for ginseng products primarily focus on ginsenoside content [2,3]. Analytical methods for quantifying LPA species require careful lipid extraction and LC–MS-based quantification due to susceptibility to hydrolysis and oxidation [50,81]. Development of validated lipidomic assays and quantitative standards for LPA-associated complexes will be essential for correlating gintonin abundance with pharmacological outcomes.

### 6.5. Clinical Translation

LPA receptor signaling has been implicated in fibrosis, tumor progression, vascular remodeling, and metabolic regulation [30,33,35]. However, clinical investigations of ginseng rarely distinguish between saponin and non-saponin fractions. Future studies employing fractionated or receptor-focused study designs may help determine whether gintonin contributes measurably to physiological outcomes.

## 7. Future Perspectives

### 7.1. Gintonin as a Prototype Phytolipid Ligand System

Gintonin represents a lipid-enriched protein complex capable of directly engaging GPCR-mediated signaling pathways [9,13]. Because GPCRs constitute one of the largest pharmacological target families [36], identification of a plant-derived ligand system targeting defined GPCR subtypes is conceptually significant. Future work should define minimal active structural units and determine receptor subtype selectivity profiles [70]. Pharmacokinetic characterization and receptor desensitization dynamics will further clarify translational potential.

### 7.2. Experimental Expansion of the Dual-Axis Framework

Membrane composition and lipid microdomain organization can influence GPCR localization and signaling efficiency [4]. Cross-talk analyses examining whether ginsenosides alter LPA receptor distribution or downstream signaling may clarify axis integration.

Systems-level approaches including phosphoproteomics and network modeling have reshaped GPCR signaling interpretation [36,38]. Such strategies may reveal coordinated pathway regulation when both axes are present.

### 7.3. Lipid Signaling in Medicinal Plants

Plant lipid signaling pathways, including phospholipase-derived messengers and oxylipin signaling systems, are increasingly recognized as key regulators of stress responses [60,61,62,63]. Systematic lipidomic profiling across medicinal plants may reveal additional receptor-active lipid assemblies.

### 7.4. Clinical and Standardization Considerations

Current regulatory frameworks emphasize ginsenoside quantification as quality markers [2]. Incorporation of lipidomic metrics may provide a more comprehensive representation of biological activity.

## 8. Conclusions

*Panax ginseng* has historically been interpreted through a ginsenoside-centric lens, with triterpenoid saponins positioned as the principal drivers of its pharmacological activity. The identification of gintonin as an LPA-enriched glycolipoprotein complex, however, introduces a mechanistically distinct receptor-targeting signaling axis that challenges this reductionist framework.

Unlike ginsenosides, which primarily modulate intracellular kinase cascades and transcriptional programs, gintonin delivers LPA species capable of engaging defined GPCR subtypes—most prominently LPA_1_ and LPA_3_—thereby initiating rapid G protein-dependent calcium mobilization and second-messenger signaling. This receptor-driven excitatory layer operates on a temporal scale fundamentally distinct from the slower transcriptional recalibration attributed to saponins. The coexistence of these modalities supports a hierarchical, temporally stratified model of ginseng pharmacology rather than biochemical redundancy.

Importantly, the translational interpretation of gintonin must be situated within the mature biological literature on the autotaxin–LPA axis, which implicates LPA receptors in fibrosis, tumor progression, vascular remodeling, immune regulation, and metabolic homeostasis. In this context, gintonin does not represent an undefined phytochemical stimulant, but a plant-derived LPA-containing signaling complex interfacing with a clinically validated GPCR network. The key pharmacological questions are therefore quantitative rather than descriptive: receptor subtype selectivity, signaling bias, ligand residence time, stability in biological matrices, and tissue-level exposure dynamics.

Nevertheless, critical structural uncertainties remain. The supramolecular architecture of gintonin has not yet been resolved at high resolution, and the stoichiometric relationship between LPA species and associated proteins remains incompletely defined. Without orthogonal structural validation, it remains unclear whether gintonin represents a discrete, evolutionarily organized lipid–protein assembly or a preparation-dependent lipid fraction with receptor-activating capacity. Resolving this ambiguity is essential for positioning gintonin as a coherent pharmacological entity rather than as an operational extraction term.

Equally unresolved is whether presentation of LPA within a protein scaffold modifies receptor conformational landscapes or signaling kinetics in a manner consistent with functional selectivity. If future studies demonstrate quantifiable differences in pathway coupling, β-arrestin recruitment, or endosomal signaling topology relative to endogenous LPA, gintonin would represent not merely a source of LPA but a presentation-modified GPCR ligand system with distinct pharmacodynamic properties.

Future progress will depend on structural resolution, quantitative receptor pharmacology, and integrative in vivo modeling of ligand stability, distribution, and signaling persistence. These efforts will clarify whether gintonin represents a structurally defined plant-derived GPCR ligand complex or a bioactive lipid formulation embedded within botanical matrices. Either outcome will refine the molecular understanding of *Panax ginseng* and situate its bioactivity within the broader framework of contemporary receptor pharmacology.

## Figures and Tables

**Table 1:** Overview of LPA receptor subtypes and major downstream signaling pathways relevant to gintonin signaling. LPA receptors couple to multiple G proteins and activate diverse signaling cascades, including PLC/IP_3_-mediated Ca^2+^ release, MAPK phosphorylation, PI3K/Akt activation, and RhoA-mediated cytoskeletal remodeling [15,16,17]. Gintonin has been primarily associated with activation of LPA_1_ and LPA_3_ subtypes [9,11], though comprehensive subtype-specific profiling remains incomplete.

Receptor Subtype	Coupled G Proteins	Major Downstream Pathways	Physiological Domains	Key Reference
** LPA_1_**	Gαi/o, Gαq, Gα12/13	PLC/IP_3_/Ca^2+^, MAPK, RhoA	CNS development, vascular regulation, immune signaling	[14,15,21]
**LPA_2_**	Gαi/o, Gαq	PI3K/Akt, MAPK	Immune cells, epithelial systems	[17,18]
**LPA_3_**	Gαi/o, Gαq	Ca^2+^ mobilization, ERK	CNS, reproductive tissues	[15,19]
**LPA_4–__6_**	Variable coupling (Gα12/13, Gαs, Gαi)	RhoA activation, cAMP modulation	Developmental processes, vascular biology	[15,16,20]

**Table 2:** Pharmacodynamic differentiation between endogenous LPA and gintonin-associated LPA: implications for signaling bias and receptor topology. Although gintonin-associated LPA shares identical chemical headgroups with endogenous LPA, ligand presentation, membrane partitioning, and kinetic parameters may influence receptor conformational landscapes and downstream pathway coupling [39,70]. Whether gintonin modifies signaling topology relative to freely diffusible LPA remains experimentally unresolved.

Pharmacological Dimension	Endogenous LPA	Gintonin-Associated LPA	Implication	Required Test	Key References
**Ligand source and presentation**	Autotaxin-generated; freely diffusible lipid mediator	Delivered within a protein-associated complex	Scaffolded delivery may alter receptor engagement geometry	Free LPA vs. intact gintonin at matched molar concentrations	[9,43]
**Membrane partitioning dynamics**	Rapid insertion into lipid bilayer	Potentially modified insertion via scaffold-mediated presentation	Altered local concentration at receptor interface; possible microdomain bias	Lipid partition assays; membrane microdomain localization studies	[55,69]
**G protein coupling profile**	Gαq, Gαi/o, Gα12/13 coupling documented	Ca^2+^ mobilization demonstrated; broader coupling unresolved	Possible pathway-selective amplification	BRET/FRET-based pathway profiling; subtype-selective assays	[15,70]
**β-arrestin recruitment**	Receptor-dependent internalization described	Not systematically examined	Potential bias toward G protein vs. arrestin signaling	β-arrestin recruitment assays; receptor trafficking studies	[40]
**Signal kinetics (onset and decay)**	Rapid synthesis and degradation via lipid phosphatases	Possible transient protection from degradation	Altered residence time may shift signaling persistence	Real-time kinetic signaling analysis; degradation profiling	[50,71]
**Endosomal vs. plasma membrane signaling**	Endosomal GPCR signaling established in multiple systems	Unknown whether scaffold modifies internalization topology	Spatial signaling bias possible	Confocal imaging; endosomal signaling biosensors	[39,71]
**Desensitization and receptor recycling**	Regulated by phosphorylation and arrestin pathways	Not characterized for gintonin	Altered desensitization may affect cumulative signaling output	Receptor internalization/recycling kinetics assays	[40]
**Subtype selectivity (LPA_1_– _6 _)**	Differential subtype expression and pharmacology established	Primarily LPA_1_/LPA_3_ implicated	Quantitative subtype bias remains undefined	Use subtype-selective expression systems; antagonists	[9,17]
**Pathophysiological modulation**	Implicated in fibrosis, cancer, vascular remodeling, metabolism	Context-dependent effects unknown	Dose, persistence, and tissue distribution critical	In vivo receptor occupancy and pharmacodynamic modeling	[30,35]

**Table 3:** Systems-level integration of the dual signaling architecture in *Panax ginseng*. The gintonin axis is characterized by rapid GPCR-mediated Ca^2+^ signaling across multiple physiological systems [9,15], whereas the ginsenoside axis primarily modulates kinase cascades and transcriptional programs over longer time scales [3,4]. This temporal and mechanistic layering may contribute to the broad pharmacological profile of ginseng.

Physiological System	Gintonin Axis (GPCR Signaling)	Ginsenoside Axis (Modulatory Signaling)	**Key Reference**
**Central Nervous System**	Ca^2+^ mobilization; neurotransmitter release	Neuroprotection; anti-inflammatory gene regulation	[3,9,11,25]
**Metabolic Tissues**	Ca^2+^-dependent metabolic signaling	Insulin sensitization; AMPK modulation	[5,15,35]
**Vascular System**	Acute Ca^2+^-dependent tone and permeability modulation	NO production; endothelial protection	[4,26,28]
**Immune System**	Rapid Ca^2+^-linked activation and migration	NF-κB and MAPK pathway suppression	[6,65,66]
**Temporal Profile**	Seconds–minutes (GPCR/second messenger kinetics)	Hours–days (transcriptional/kinase reprogramming)	[3,14,38]

**Table 4:** Structural definition gaps and experimental priorities for establishing gintonin as a receptor-active lipid–protein assembly. Gintonin has been described as an LPA-enriched glycolipoprotein complex based on chromatographic fractionation, lipidomic profiling, and proteomic identification [9,42]. However, key structural parameters that define a coherent “ligand system” remain unresolved, and extraction-driven heterogeneity cannot be excluded [55,81].

Structural Parameter (What Must Be Defined)	Critical Uncertainty/Alternative Explanation	Why This Matters Pharmacologically	Priority Experiments (Actionable)	Key Reference
**Molecular identity: discrete assembly vs. heterogeneous mixture**	Activity may arise from (i) a reproducible supramolecular complex, (ii) a heterogeneous set of LPA-associated proteins, or (iii) an extraction-derived lipid–protein aggregate	If not discrete/reproducible, “gintonin” acts as *variable LPA delivery* rather than a defined ligand entity → subtype selectivity, potency, and reproducibility claims weaken	Orthogonal structural validation across batches: (1) native fractionation + lipidomics, (2) replicate preparations with standardized workflow, (3) batch-to-batch variance statistics	[9,42,55,81]
**Stoichiometry (LPA:protein ratio) and composition map**	Unknown LPA:protein stoichiometry; unknown whether LPA content scales linearly with activity; unclear if minor LPA species drive potency	Without stoichiometry, EC_50_ comparisons and “high-affinity” narrative can be confounded by effective free LPA concentration	Native MS for intact mass distribution + absolute quantitative lipidomics with internal standards; report LPA molecules per protein complex (distribution, not just mean)	[9,50,81]
**Minimal receptor-active unit**	Minimal unit could be: (a) protein-bound LPA, (b) micelle-like aggregates, or (c) free LPA released upon dilution	Determines whether the “protein scaffold” is mechanistically essential or incidental	Activity-guided disassembly/reconstitution: (1) controlled dissociation series, (2) reconstitution with purified candidate proteins + defined LPA species, (3) compare to matched free LPA dose–response	[9,10,55]
**Binding mode: defined lipid-binding pocket vs. nonspecific hydrophobic association**	Could be nonspecific adsorption during extraction rather than evolved pockets; “complex” may be loosely organized	Pocketed binding implies constrained presentation → supports mechanistic claims about altered kinetics/bias; nonspecific association implies formulation artifact	HDX-MS or limited proteolysis mapping + cross-linking MS (XL-MS) to detect specific interfaces; targeted mutational/competition assays if a dominant scaffold protein is identified	[57,58,59]
**Physical stability under physiological conditions** (ionic strength, albumin, bile salts, serum lipases)	Complex may dissociate rapidly in biologic matrices; LPA transfer to albumin/lipoproteins may dominate in vivo	If unstable, in vivo effects may reflect systemic LPA handling rather than scaffolded presentation	Stability panel: incubate in serum/albumin/bile conditions → measure (1) free vs. bound LPA fraction, (2) persistence of Ca^2+^ signal, (3) lipid degradation products	[50,81]
**Extraction/processing dependence (artifact risk)**	Solvent exposure can drive aggregation and lipid exchange → “gintonin” may be preparation-conditional	If preparation-dependent, standardization and inter-study comparability become major barriers	Side-by-side comparison of extraction protocols + QC fingerprinting (lipidomics/proteomics) linked to bioassay potency	[42,55]
**Structural basis for GPCR pharmacology claims** (e.g., “high affinity”, subtype preference)	Without a defined ligand entity, receptor pharmacology parameters (affinity, efficacy) may reflect kinetic/partition effects not captured by classical assumptions	Impacts interpretation of subtype selectivity and “ligand system” framing	Receptor pharmacology with defined materials: measure kinetics (k_on/k_off), concentration normalization to LPA molarity, and compare to matched free LPA	[38,70]
**Suitability of high-resolution structural work** (cryo-EM, integrative modeling feasibility)	Heterogeneity may preclude cryo-EM unless stabilized/monodisperse	Sets realistic roadmap: “structure-first” vs. “composition-first”	Stepwise roadmap: (1) monodispersity screening (SEC-MALS/DLS), (2) native MS to assess heterogeneity, then (3) cryo-EM/XL-MS integration if tractable	[38]

**Abbreviations:** HDX-MS, hydrogen–deuterium exchange mass spectrometry; XL-MS, cross-linking mass spectrometry; SEC-MALS, size-exclusion chromatography coupled with multi-angle light scattering; DLS, dynamic light scattering.

## Data Availability

No new data were created or analyzed in this study. Data sharing is not applicable to this review.

## Abbreviations

Akt	Protein kinase B
AMPK	AMP-activated protein kinase
ATX	Autotaxin
BRET	Bioluminescence resonance energy transfer
Ca^2+^	Calcium ion
CNS	Central nervous system
DLS	Dynamic light scattering
EC_50_	Half maximal effective concentration
EDG	Endothelial differentiation gene
ER	Endoplasmic reticulum
ERK	Extracellular signal-regulated kinase
FRET	Förster resonance energy transfer
GPCR	G protein-coupled receptor
Gαi/o	G alpha inhibitory/o
Gαq/11	G alpha q/11
Gα12/13	G alpha 12/13
HDX-MS	Hydrogen–deuterium exchange mass spectrometry
IP_3_	Inositol 1,4,5-trisphosphate
LPA	Lysophosphatidic acid
LPAR	Lysophosphatidic acid receptor gene
LPA_1_–LPA_6_	Lysophosphatidic acid receptor subtypes 1–6
LC–MS	Liquid chromatography–mass spectrometry
MAPK	Mitogen-activated protein kinase
NF-κB	Nuclear factor kappa B
NO	Nitric oxide
nsLTP	Nonspecific lipid transfer protein
PI3K	Phosphoinositide 3-kinase
PLC	Phospholipase C
RhoA	Ras homolog family member A
SEC-MALS	Size-exclusion chromatography coupled with multi-angle light scattering
XL-MS	Cross-linking mass spectrometry

## References

[1] Shi Z.Y., Zeng J.Z., Wong A.S.T. (2019). Chemical structures and pharmacological profiles of ginseng saponins. Molecules.

[2] Leung K.W., Wong A.S. (2010). Pharmacology of ginsenosides: A literature review. Chin. Med..

[3] Ratan Z.A., Haidere M.F., Hong Y.H., Park S.H., Lee J.O., Lee J., Cho J.Y. (2021). Pharmacological potential of ginseng and its major component ginsenosides. J. Ginseng Res..

[4] Verstraeten S.L., Lorent J.H., Mingeot-Leclercq M.P. (2020). Lipid Membranes as key targets for the pharmacological actions of ginsenosides. Front. Pharmacol..

[5] Ghafouri-Fard S., Balaei N., Shoorei H., Hasan S.M.F., Hussen B.M., Talebi S.F., Taheri M., Ayatollahi S.A. (2022). The effects of ginsenosides on PI3K/AKT signaling pathway. Mol. Biol. Rep..

[6] Jang W.Y., Hwang J.Y., Cho J.Y. (2023). Ginsenosides from Panax ginseng as key modulators of NF-κB signaling are powerful anti-inflammatory and anticancer agents. Int. J. Mol. Sci..

[7] Moolenaar W.H. (1999). Bioactive lysophospholipids and their G protein-coupled receptors. Exp. Cell Res..

[8] Radeff-Huang J., Seasholtz T.M., Matteo R.G., Brown J.H. (2004). G protein mediated signaling pathways in lysophospholipid induced cell proliferation and survival. J. Cell Biochem..

[9] Hwang S.H., Shin T.J., Choi S.H., Cho H.J., Lee B.H., Pyo M.K., Lee J.H., Kang J., Kim H.J., Park C.W. (2012). Gintonin, newly identified compounds from ginseng, is novel lysophosphatidic acids-protein complexes and activates G protein-coupled lysophosphatidic acid receptors with high affinity. Mol. Cells.

[10] Nah S.Y. (2012). Gintonin: A novel ginseng-derived ligand that targets G protein-coupled lysophosphatidic acid receptors. Curr. Drug Targets.

[11] Kim H., Lee B.H., Choi S.H., Kim H.J., Jung S.W., Hwang S.H., Rhim H., Kim H.C., Cho I.H., Nah S.Y. (2015). Gintonin stimulates gliotransmitter release in cortical primary astrocytes. Neurosci. Lett..

[12] Yung Y.C., Stoddard N.C., Chun J. (2014). LPA receptor signaling: Pharmacology, physiology, and pathophysiology. J. Lipid Res..

[13] Im D.S., Nah S.Y. (2013). Yin and Yang of ginseng pharmacology: Ginsenosides vs gintonin. Acta Pharmacol. Sin..

[14] Fukushima N., Kimura Y., Chun J. (1998). A single receptor encoded by vzg-1/lpA1/edg-2 couples to G proteins and mediates multiple cellular responses to lysophosphatidic acid. Proc. Natl. Acad. Sci. USA.

[15] Noguchi K., Herr D., Mutoh T., Chun J. (2009). Lysophosphatidic acid (LPA) and its receptors. Curr. Opin. Pharmacol..

[16] Kihara Y., Maceyka M., Spiegel S., Chun J. (2014). Lysophospholipid receptor nomenclature review: IUPHAR Review 8. Br. J. Pharmacol..

[17] Choi J.W., Herr D.R., Noguchi K., Yung Y.C., Lee C.W., Mutoh T., Lin M.E., Teo S.T., Park K.E., Mosley A.N. (2010). LPA receptors: Subtypes and biological actions. Annu. Rev. Pharmacol. Toxicol..

[18] Bandoh K., Aoki J., Hosono H., Kobayashi S., Kobayashi T., Murakami-Murofushi K., Tsujimoto M., Arai H., Inoue K. (1999). Molecular cloning and characterization of a novel human G-protein-coupled receptor, EDG7, for lysophosphatidic acid. J. Biol. Chem..

[19] Lee C.W., Rivera R., Gardell S., Dubin A.E., Chun J. (2006). GPR92 as a new G12/13- and Gq-coupled lysophosphatidic acid receptor that increases cAMP, LPA5. J. Biol. Chem..

[20] Yanagida K., Masago K., Nakanishi H., Kihara Y., Hamano F., Tajima Y., Taguchi R., Shimizu T., Ishii S. (2009). Identification and characterization of a novel lysophosphatidic acid receptor, p2y5/LPA6. J. Biol. Chem..

[21] Contos J.J., Fukushima N., Weiner J.A., Kaushal D., Chun J. (2000). Requirement for the lpA1 lysophosphatidic acid receptor gene in normal suckling behavior. Proc. Natl. Acad. Sci. USA.

[22] Ueda H. (2020). LPA receptor signaling as a therapeutic target for radical treatment of neuropathic pain and fibromyalgia. Pain Manag..

[23] Engelbrecht E., MacRae C.A., Hla T. (2021). Lysolipids in vascular development, biology, and disease. Arterioscler. Thromb. Vasc. Biol..

[24] Hemmings D.G., Brindley D.N. (2020). Signalling by lysophosphatidate and its health implications. Essays Biochem..

[25] Weiner J.A., Hecht J.H., Chun J. (1998). Lysophosphatidic acid receptor gene vzg-1/lpA1/edg-2 is expressed by mature oligodendrocytes during myelination in the postnatal murine brain. J. Comp. Neurol..

[26] van Nieuw Amerongen G.P., Vermeer M.A., van Hinsbergh V.W. (2000). Role of RhoA and Rho kinase in lysophosphatidic acid-induced endothelial barrier dysfunction. Arterioscler. Thromb. Vasc. Biol..

[27] Ren Y., Guo L., Tang X., Apparsundaram S., Kitson C., Deguzman J., Fuentes M.E., Coyle L., Majmudar R., Allard J. (2013). Comparing the differential effects of LPA on the barrier function of human pulmonary endothelial cells. Microvasc. Res..

[28] Siess W., Zangl K.J., Essler M., Bauer M., Brandl R., Corrinth C., Bittman R., Tigyi G., Aepfelbacher M. (1999). Lysophosphatidic acid mediates the rapid activation of platelets and endothelial cells by mildly oxidized low density lipoprotein and accumulates in human atherosclerotic lesions. Proc. Natl. Acad. Sci. USA.

[29] Pamuklar Z., Federico L., Liu S., Umezu-Goto M., Dong A., Panchatcharam M., Fulkerson Z., Berdyshev E., Natarajan V., Fang X. (2009). Autotaxin/lysopholipase D and lysophosphatidic acid regulate murine hemostasis and thrombosis. J. Biol. Chem..

[30] Tager A.M., LaCamera P., Shea B.S., Campanella G.S., Selman M., Zhao Z., Polosukhin V., Wain J., Karimi-Shah B.A., Kim N.D. (2008). The lysophosphatidic acid receptor LPA1 links pulmonary fibrosis to lung injury by mediating fibroblast recruitment and vascular leak. Nat. Med..

[31] Funke M., Zhao Z., Xu Y., Chun J., Tager A.M. (2012). The lysophosphatidic acid receptor LPA1 promotes epithelial cell apoptosis after lung injury. Am. J. Respir. Cell Mol. Biol..

[32] Ren J., Xiao Y.J., Singh L.S., Zhao X., Zhao Z., Feng L., Rose T.M., Prestwich G.D., Xu Y. (2006). Lysophosphatidic acid is constitutively produced by human peritoneal mesothelial cells and enhances adhesion, migration, and invasion of ovarian cancer cells. Cancer Res..

[33] Yu S., Murph M.M., Lu Y., Liu S., Hall H.S., Liu J., Stephens C., Fang X., Mills G.B. (2008). Lysophosphatidic acid receptors determine tumorigenicity and aggressiveness of ovarian cancer cells. J. Natl. Cancer Inst..

[34] Valdés-Rives S.A., González-Arenas A. (2017). Autotaxin-lysophosphatidic acid: From inflammation to cancer development. Mediat. Inflamm..

[35] Federico L., Ren H., Mueller P.A., Wu T., Liu S., Popovic J., Blalock E.M., Sunkara M., Ovaa H., Albers H.M. (2012). Autotaxin and its product lysophosphatidic acid suppress brown adipose differentiation and promote diet-induced obesity in mice. Mol. Endocrinol..

[36] Hauser A.S., Attwood M.M., Rask-Andersen M., Schiöth H.B., Gloriam D.E. (2017). Trends in GPCR drug discovery: New agents, targets and indications. Nat. Rev. Drug Discov..

[37] Congreve M., Marshall F. (2010). The impact of GPCR structures on pharmacology and structure-based drug design. Br. J. Pharmacol..

[38] Rasmussen S.G., DeVree B.T., Zou Y., Kruse A.C., Chung K.Y., Kobilka T.S., Thian F.S., Chae P.S., Pardon E., Calinski D. (2011). Crystal structure of the beta2 adrenergic receptor-Gs protein complex. Nature.

[39] Wootten D., Christopoulos A., Marti-Solano M., Babu M.M., Sexton P.M. (2018). Mechanisms of signalling and biased agonism in G protein-coupled receptors. Nat. Rev. Mol. Cell Biol..

[40] Shenoy S.K., Lefkowitz R.J. (2011). beta-Arrestin-mediated receptor trafficking and signal transduction. Trends Pharmacol. Sci..

[41] Chrencik J.E., Roth C.B., Terakado M., Kurata H., Omi R., Kihara Y., Warshaviak D., Nakade S., Asmar-Rovira G., Mileni M. (2015). Crystal Structure of Antagonist Bound Human Lysophosphatidic Acid Receptor 1. Cell.

[42] Pyo M.K., Choi S.H., Shin T.J., Hwang S.H., Lee B.H., Kang J., Kim H.J., Lee S.H., Nah S.Y. (2011). A simple method for the preparation of crude gintonin from ginseng root, stem, and leaf. J. Ginseng Res..

[43] Umezu-Goto M., Kishi Y., Taira A., Hama K., Dohmae N., Takio K., Yamori T., Mills G.B., Inoue K., Aoki J. (2002). Autotaxin has lysophospholipase D activity leading to tumor cell growth and motility by lysophosphatidic acid production. J. Cell Biol..

[44] Tokumura A., Majima E., Kariya Y., Tominaga K., Kogure K., Yasuda K., Fukuzawa K. (2002). Identification of human plasma lysophospholipase D, a lysophosphatidic acid-producing enzyme, as autotaxin, a multifunctional phosphodiesterase. J. Biol. Chem..

[45] Xie Y., Meier K.E. (2004). Lysophospholipase D and its role in LPA production. Cell Signal..

[46] Nishimasu H., Okudaira S., Hama K., Mihara E., Dohmae N., Inoue A., Ishitani R., Takagi J., Aoki J., Nureki O. (2011). Crystal structure of autotaxin and insight into GPCR activation by lipid mediators. Nat. Struct. Mol. Biol..

[47] Kato K., Ikeda H., Miyakawa S., Futakawa S., Nonaka Y., Fujiwara M., Okudaira S., Kano K., Aoki J., Morita J. (2016). Structural basis for specific inhibition of autotaxin by a DNA aptamer. Nat. Struct. Mol. Biol..

[48] Sciorra V.A., Morris A.J. (2002). Roles for lipid phosphate phosphatases in regulation of cellular signaling. Biochim. Biophys. Acta.

[49] Brindley D.N., Pilquil C. (2009). Lipid phosphate phosphatases and signaling. J. Lipid Res..

[50] Tang X., Benesch M.G., Brindley D.N. (2015). Lipid phosphate phosphatases and their roles in mammalian physiology and pathology. J. Lipid Res..

[51] Magkrioti C., Kaffe E., Aidinis V. (2023). The role of autotaxin and LPA signaling in embryonic development, pathophysiology and cancer. Int. J. Mol. Sci..

[52] Drosouni A., Panagopoulou M., Aidinis V., Chatzaki E. (2022). Autotaxin in breast cancer: Role, epigenetic regulation and clinical implications. Cancers.

[53] Federico L., Pamuklar Z., Smyth S.S., Morris A.J. (2008). Therapeutic potential of autotaxin/lysophospholipase d inhibitors. Curr. Drug Targets.

[54] Tan Z., Lei H., Guo M., Chen Y., Zhai X. (2021). An updated patent review of autotaxin inhibitors (2017-present). Expert Opin. Ther. Pat..

[55] Tigyi G., Parrill A.L. (2003). Molecular mechanisms of lysophosphatidic acid action. Prog. Lipid Res..

[56] Noguchi K., Ishii S., Shimizu T. (2003). Identification of p2y9/GPR23 as a novel G protein-coupled receptor for lysophosphatidic acid, structurally distant from the Edg family. J. Biol. Chem..

[57] Charvolin D., Douliez J.P., Marion D., Cohen-Addad C., Pebay-Peyroula E. (1999). The crystal structure of a wheat nonspecific lipid transfer protein (ns-LTP1) complexed with two molecules of phospholipid at 2.1 A resolution. Eur. J. Biochem..

[58] Han G.W., Lee J.Y., Song H.K., Chang C., Min K., Moon J., Shin D.H., Kopka M.L., Sawaya M.R., Yuan H.S. (2001). Structural basis of non-specific lipid binding in maize lipid-transfer protein complexes revealed by high-resolution X-ray crystallography. J. Mol. Biol..

[59] van Rooijen G.J., Moloney M.M. (1995). Structural requirements of oleosin domains for subcellular targeting to the oil body. Plant Physiol..

[60] Laxalt A.M., Munnik T. (2002). Phospholipid signalling in plant defence. Curr. Opin. Plant Biol..

[61] Seo J., Lee H.Y., Choi H., Choi Y., Lee Y., Kim Y.W., Ryu S.B., Lee Y. (2008). Phospholipase A2beta mediates light-induced stomatal opening in Arabidopsis. J. Exp. Bot..

[62] Kim H.J., Ok S.H., Bahn S.C., Jang J., Oh S.A., Park S.K., Twell D., Ryu S.B., Shin J.S. (2011). Endoplasmic reticulum- and Golgi-localized phospholipase A2 plays critical roles in Arabidopsis pollen development and germination. Plant Cell.

[63] Meijer H.J., van Himbergen J.A., Musgrave A., Munnik T. (2017). Acclimation to salt modifies the activation of several osmotic stress-activated lipid signalling pathways in Chlamydomonas. Phytochemistry.

[64] Scheurer S., Schülke S. (2018). Interaction of non-specific lipid-transfer proteins with plant-derived lipids and its impact on allergic sensitization. Front. Immunol..

[65] Goetzl E.J., Kong Y., Voice J.K. (2000). Cutting edge: Differential constitutive expression of functional receptors for lysophosphatidic acid by human blood lymphocytes. J. Immunol..

[66] Fueller M., Wang D.A., Tigyi G., Siess W. (2003). Activation of human monocytic cells by lysophosphatidic acid and sphingosine-1-phosphate. Cell Signal..

[67] Hama K., Aoki J., Inoue A., Endo T., Amano T., Motoki R., Kanai M., Ye X., Chun J., Matsuki N. (2007). Embryo spacing and implantation timing are differentially regulated by LPA3-mediated lysophosphatidic acid signaling in mice. Biol. Reprod..

[68] Isogai S., Deupi X., Opitz C., Heydenreich F.M., Tsai C.J., Brueckner F., Schertler G.F., Veprintsev D.B., Grzesiek S. (2016). Backbone NMR reveals allosteric signal transduction networks in the beta1-adrenergic receptor. Nature.

[69] Milligan G., Ramsay D., Pascal G., Carrillo J.J. (2003). GPCR dimerisation. Life Sci..

[70] Kenakin T., Christopoulos A. (2013). Signalling bias in new drug discovery: Detection, quantification and therapeutic impact. Nat. Rev. Drug Discov..

[71] Irannejad R., Tomshine J.C., Tomshine J.R., Chevalier M., Mahoney J.P., Steyaert J., Rasmussen S.G., Sunahara R.K., El-Samad H., Huang B. (2013). Conformational biosensors reveal GPCR signalling from endosomes. Nature.

[72] Tang N., Zhao Y., Feng R., Liu Y., Wang S., Wei W., Ding Q., An M.S., Wen J., Li L. (2014). Lysophosphatidic acid accelerates lung fibrosis by inducing differentiation of mesenchymal stem cells into myofibroblasts. J. Cell Mol. Med..

[73] Kim K.S., Sengupta S., Berk M., Kwak Y.G., Escobar P.F., Belinson J., Mok S.C., Xu Y. (2006). Hypoxia enhances lysophosphatidic acid responsiveness in ovarian cancer cells and lysophosphatidic acid induces ovarian tumor metastasis in vivo. Cancer Res..

[74] Lin S., Wang D., Iyer S., Ghaleb A.M., Shim H., Yang V.W., Chun J., Yun C.C. (2009). The absence of LPA2 attenuates tumor formation in an experimental model of colitis-associated cancer. Gastroenterology.

[75] Wang F.Q., Barfield E., Dutta S., Pua T., Fishman D.A. (2009). VEGFR-2 silencing by small interference RNA (siRNA) suppresses LPA-induced epithelial ovarian cancer (EOC) invasion. Gynecol. Oncol..

[76] Masago K., Kihara Y., Yanagida K., Hamano F., Nakagawa S., Niwa M., Shimizu T. (2018). Lysophosphatidic acid receptor, LPA(6), regulates endothelial blood-brain barrier function: Implication for hepatic encephalopathy. Biochem. Biophys. Res. Commun..

[77] Cummings R., Zhao Y., Jacoby D., Spannhake E.W., Ohba M., Garcia J.G., Watkins T., He D., Saatian B., Natarajan V. (2004). Protein kinase Cdelta mediates lysophosphatidic acid-induced NF-kappaB activation and interleukin-8 secretion in human bronchial epithelial cells. J. Biol. Chem..

[78] Hoeglund A.B., Howard A.L., Wanjala I.W., Pham T.C., Parrill A.L., Baker D.L. (2010). Characterization of non-lipid autotaxin inhibitors. Bioorg. Med. Chem..

[79] Vilardaga J.P. (2010). Theme and variations on kinetics of GPCR activation/deactivation. J. Recept. Signal Transduct. Res..

[80] Heck J., Parutto P., Ciuraszkiewicz A., Bikbaev A., Freund R., Mitlöhner J., Andres-Alonso M., Fejtova A., Holcman D., Heine M. (2019). Transient confinement of Ca_V_2.1 Ca^2+^-channel splice variants shapes synaptic short-term plasticity. Neuron.

[81] Okudaira S., Yukiura H., Aoki J. (2010). Biological roles of lysophosphatidic acid signaling through its production by autotaxin. Biochimie.

