# Correction: Kim, K.-H.; Yoo, B.C. Gintonin as a Lysophosphatidic Acid-Enriched GPCR Ligand System: Molecular Architecture and Receptor Pharmacology in *Panax ginseng*. *Biomolecules* 2026, *16*, 465

**DOI:** 10.3390/biom16060779

**Published:** 2026-05-26

**Authors:** Kyung-Hee Kim, Byong Chul Yoo

**Affiliations:** 1 Department of Applied Chemistry, School of Science and Technology, Kookmin University, Seoul 02707, Republic of Korea; kyungheekim@kookmin.ac.kr; 2 Antibody Research Institute, Kookmin University, Seoul 02707, Republic of Korea; 3 Diagnostic Research Team, InnoBation Bio R&D Center, Seoul 03929, Republic of Korea

In the published [1] article, several statements describing the composition of lysophosphatidic acid (LPA) species in the gintonin fraction require clarification.

Originally, the manuscript stated that major LPA species include “C16:0, C18:0, and C18:1”. While this description reflects previously reported analytical findings [9,42], it may not fully represent the current consensus regarding relative abundance.

Recent interpretations suggest that unsaturated LPA species, particularly C18:2, are predominant in gintonin preparations, whereas saturated species such as C18:0 may be present at lower levels.

Accordingly, the following corrections have been made:

Correction in Abstract

Original:

Biochemical analyses have identified major LPA species within the gintonin fraction, including C16:0, C18:0, and C18:1, stabilized within a proteinaceous matrix that may influence receptor engagement kinetics.

Corrected:

Biochemical analyses have identified major LPA species within the gintonin fraction, including C16:0, C18:1, and other unsaturated LPA species such as C18:2 stabilized within a proteinaceous matrix that may influence receptor engagement kinetics.

Correction in Introduction

Original:

Biochemical analyses have demonstrated that the gintonin fraction contains specific LPA molecular species, including C16:0, C18:0, and C18:1, stabilized within a proteinaceous scaffold derived from ginseng tissue [9,42].

Corrected:

Biochemical analyses have demonstrated that the gintonin fraction contains specific LPA molecular species, including C16:0 and C18:1, and unsaturated species such as C18:2, stabilized within a proteinaceous scaffold derived from ginseng tissue [9,42].

Correction in Section 2.2

Original:

Mass spectrometric analyses of the gintonin-enriched fraction revealed the presence of multiple LPA molecular species, predominantly C16:0, C18:0, and C18:1 [9].

Corrected:

Mass spectrometric analyses of the gintonin-enriched fraction revealed the presence of multiple LPA molecular species, comprising multiple LPA species, including C16:0 and C18:1, and unsaturated species such as C18:2, which are considered predominant in recent analyses [9].

The authors state that the scientific conclusions are unaffected. This correction was approved by the Academic Editor. The original publication has also been updated.
